# Erectile dysfunction and associated factors among patients with diabetes attending follow-up at a public hospital, Harar, Eastern Ethiopia. A cross-sectional study design

**DOI:** 10.3389/fendo.2023.1131555

**Published:** 2023-06-13

**Authors:** Matebu Bekele Gobena, Tekabe Abdosh, Merga Dheresa, Deribe Bekele Dechasa

**Affiliations:** ^1^ Department of Internal Medicine, School of Medicine, Collage of Medicine and Health sciences, Dire Dawa University, Dire Dawa, Ethiopia; ^2^ Department of Internal Medicine, School of Medicine, Collage of Health and Medical Sciences, Haramaya University, Harar, Ethiopia; ^3^ School of Nursing and Midwifery, Collage of Health and Medical Sciences, Haramaya University, Harar, Ethiopia

**Keywords:** magnitude, erectile dysfunction diabetes mellitus, Eastern Ethiopia, complication of diabetes, patient

## Abstract

**Background:**

The global prevalence of erectile dysfunction among patients with diabetes is high. It is the most underestimated problem but has a great physical, psychological, and social impact on the individual with the disease, family, and society in general. Thus, this study aimed to assess the magnitude of erectile dysfunction and associated factors among patients with diabetes attending follow-up at a public hospital, Harar, Eastern Ethiopia.

**Methods:**

Facility-based cross-sectional study was conducted on selected 210 adult male patients with diabetes attending follow-up at a public hospital, Harar, Eastern Ethiopia, from 1 February to 30 March 2020. Simple random sampling was used to select study participants. A pre-tested interviewer-administered structured questionnaire was used to collect the data. The data were entered to EpiData version 3.1 and exported to SPSS version 20 for analysis. Bivariate and multivariable binary logistic regression were carried out, and a P-value of <0.05 was taken as statistically significant.

**Result:**

A total of 210 adult male patients with diabetes participated in the study. The overall magnitude of erectile dysfunction was 83.8%, with 26.7% suffering from mild, 37.5% mild to moderate, 29% moderate, and 6.8% severe erectile dysfunctions. Age 46–59 years [adjusted odds ratio (AOR): 2.560; 95% confidence interval (CI) (1.73, 6.53)], age ≥ 60 years [AOR: 2.9; 95% CI (1.48, 5.67)], and poor glycemic control [AOR: 2.140; 95% CI (1.9, 7.44)] were significantly associated with erectile dysfunction among patients with diabetes.

**Conclusion:**

The present study revealed a high magnitude of erectile dysfunction among population with diabetes. The age categories of 46–59 and ≥60 and having poor glycemic control were the only variables significantly associated with erectile dysfunction. Thus, routine screening and management for erectile dysfunction in patients with diabetes should be part of routine medical care particularly for adult male patients and those with poor glycemic control.

## Introduction

Diabetes mellitus (DM) is one of the most common and serious non-communicable disease affecting the lives and wellbeing of individuals with the disease, families, and societies at large. In 2019, approximately 463 million (9.3%) people were living with diabetes, and it was estimated that, by 2045, the prevalence will rise to 10.9% (700 million) (1). Its complications have major and long-lasting impacts at different levels (2). One of the most common and underestimated complications among DM is erectile dysfunction (ED) (3). According to the National Institutes of Health Consensus Development Conference, ED is defined as the persistent inability to achieve or maintain an erection sufficient for satisfactory sexual performance (4). ED is two to three times more common among individuals with DM than those without DM (5, 6).

The global prevalence of ED was 3%–76.5% (7). There is a great variation in the prevalence of ED among male patients with DM. On the basis of different epidemiological data, its prevalence was 52% (3). Different studies were carried out in different parts of the world, where the prevalence of ED among patients with DM ranges from 6.8% in Ethiopia to 95% in South Africa (8–21). In Africa, its general prevalence among patients with DM was 71.45% (22). Different studies on the prevalence of ED among patients with DM in some parts Ethiopia were carried out, and the prevalence was from 6.8% to 85.5% (8, 11, 19, 21, 23–25).

Although the number of people living with diabetes is rising in Ethiopia, there are only few studies on the complications of DM particularly ED. ED has a significant impact on the individual both physically and psychologically. It can also impair the quality of life of the patients, as well as their partners and families in general (5, 26). Factors including depression, older age, low educational status, poor quality of life, lack of regular physical activity, longer duration of DM, history of cardiovascular disease, cigarette smoking, hypercholesterolemia, poor glycemic control, obesity, taking beta blockers, and comorbidity were stated as the determinant factors for ED in patients with DM in different studies (8, 9, 11, 13, 16–19, 21, 23–25, 27).

ED is a treatable condition. If effective care and management including lifestyle modification, psychosexual therapy, and pharmacotherapy is applied, then it can be cured for up to 95% of cases (28). However, talking about sexual practice and experience is a very sensitive issue in Ethiopia where it is considered as a shameful act. This makes the diagnosis and treatment of ED difficult. The poor culture of openly discussing the sexual health problem among the community may mask the real magnitude of the problem in the population with diabetes. No study on ED in the eastern part of the country was carried out, and knowing its current magnitude and determinant factors may be important for early detection, managing the problem, and improving the quality of life of the patients. Thus this study aimed to assess the magnitude of ED and associated factors among patients with diabetes attending follow-up at a public hospital, Harar, Eastern Ethiopia.

## Materials and methods

### Study setting and period

A facility-based cross-sectional study was conducted at HiwotFana Comprehensive Specialized Hospital (HFCSH) from 1 February to 30 March 2020. The hospital is found in Harar city, Harari region, which is found at a distance of 526 km southeast of the capital city, Addis Ababa. HFCSH is a teaching hospital with a catchment of 5.2 million populations. It has a total of 185 inpatient beds distributed among four major departments.

### Study population

All adult male patients with diabetes on follow-up at HFCSH during the study period were the source population, and male patients with diabetes who were on follow-up clinic during the study period, who are ≥ 18 years of age, and who are willing to participate in the study were included in the study. However, patients who were critically ill, have serious mental illness, had paraplegia from any cause, had past lower urinary tract and prostate surgery, and were not sexually active not because of ED for the previous 6 months prior to the study were excluded from the study.

### Sample size determination and sampling procedure

The sample size was determined using the single population estimation formula by considering margin of error of 5%, confidence level of 95%, and the proportion of ED among patients with DM of 85.5% (24). The calculated sample size was 191, and, by adding 10% non-response rate, the final sample size became 210. To select the study participants, the number of male patients with diabetes expected to have follow-up during the study period in the follow-up clinic was taken from follow-up registration book; then, a systematic random sampling method was used to select study participants.

### Data collection tools and method

The data were collected by using an interviewer-administered structured questionnaire. The questionnaire was first prepared in English and then translated to Afan Oromo and Amharic languages and then translated back to English by language experts to check for consistency. The questionnaire was taken from previous similar studies in Ethiopia and from the abridged five-item version of the International Index of Erectile Function (IIEF-5) (29). The instrument produced high sensitivity and specificity, 92.2 and 92.1%, respectively. It has a total of five questions scored out of 25. Accordingly, individuals who scored 1–21 were reported as having ED. While those who scored 22–25 were reported as not having ED. Those who scored 1–7, 8–11, 12–16, and 17–21 out of 25 points were classified as having severe ED, moderate ED, mild to moderate ED, and mild ED, respectively (29). Four medical intern students collected the data.

### Data quality control

The quality of data was assured by proper designing of the questionnaire and pre-testing on 10% of the total sample size before 1 week of the actual data collection, and amendments were made based on the information obtained. One-day training was given to the data collectors and supervisors by the principal investigator. During data collection, each questionnaire was reviewed for completeness and consistency by supervisor, and all the necessary feedback was given to the data collectors immediately.

### Data processing and analysis

The collected data were first checked for its completeness, cleaned and entered into EpiData version 3.1, and then exported to SPSS version 20 for analysis. Descriptive statistics such as frequency, percentage, mean, and standard deviation (SD) were computed. Bivariate binary logistic regression analysis was carried out to determine the association between each independent variable and dependent variable. Variables with p < 0.2 in bivariate analysis were entered for multiple logistic regressions. Finally, binary logistic regression was carried out, and a P-value of <0.05 was used to declare the statistical significance. To measure the strength of association, adjusted odds ratio (AOR) with their corresponding 95% confidence interval (CI) was used. The fitness of the model was checked using Hosmer and Lemeshow test, and multicollinearity was checked by using variance inflation factor of <10 and tolerance of >0.2.

## Result

### Sociodemographic characteristics of the study participants

A total of 210 adult male patients with diabetes participated in the study with a 100% response rate. The mean age of the study participants was 54.53 (± 13.73 SD) with a majority (79; 37.6%) of the study participants in the age group between 46 and 59 years. From the total participants, a majority (197; 93.8%) of them were married. Regarding their educational status, 86 (41%) had elementary education and 80 (40.5%) were government employees. Almost two-thirds (142; 67.6%) of the respondents, had an average monthly income of 1,501–3,000 Ethiopian birr (**Table 1**).

**Table 1:** Sociodemographic characteristics of adult male patients with diabetes attending follow-up at Hiwot Fana Comprehensive Specialized University Hospital, Harar, Eastern Ethiopia, 2020 (n = 210).

Variable	Frequency	Percentage (%)
Age (years)		
18–30	8	3.8
31–45	50	23.8
46–59	79	37.6
≥60	73	34.8
Marital status		
Married	197	93.8
Others*	13	6.2
Educational status		
Illiterate	33	15.7
Elementary school (1–8)	86	41.0
High school (9–12)	55	26.2
College and above	36	17.1
Occupational status		
Government employed	85	40.5
Farmer	53	25.2
Merchant	53	25.2
Others**	19	9.0
Income		
<1,500 ETB	21	10.0
1,501–3,000 ETB	142	67.6
>3,000 ETB	47	22.4

ETB, Ethiopian birr; Other*: single, widowed, and divorced; Others**: private employee, no occupation, and retired.

### Behavioral characteristics of the study participants

**Figure 1:**
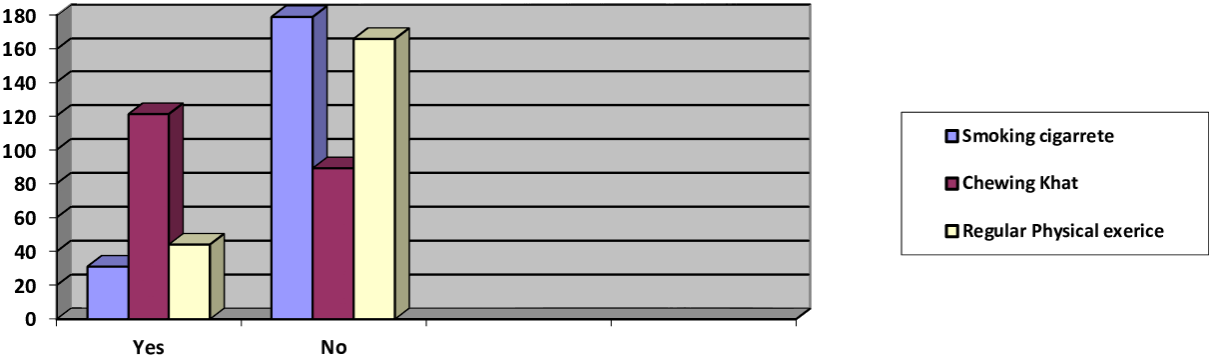
Behavioral characteristics of male patients with diabetes attending diabetic clinic at Hiwot Fana Comprehensive Specialized University Hospital (N = 210), Harar, Eastern Ethiopia, 2020 (n = 210).

From the total participants, almost all of them did not have unsafe alcohol consumption. Majority of them (121; 57.6%) chew khat, and 32 (15.2%) were smokers. Regarding physical exercise, only about one-fifth (44; 21%) of the participants had regular physical exercise (**Figure 1**).

### Medical characteristics of the study participants

Inadequate glycemic control was present at the time of the investigation, as evidenced by the mean fasting blood sugar (FBS) of 162 mg/dl (+19.9 SD). The mean body mass index (BMI) of the participants was 24.6 kg/m^2^ ( **±** 3.3 SD), and almost half of them (103; 49%) had a BMI of 18.5 to 24.96 kg/m^2^. Majority (54.3%) of the respondents lived with diabetes for < 5 years with mean duration of 5.8 (**±** 4.21 SD) years. Majority of the participants (117; 55.7%) were within normal range of blood pressure (<140/90). Almost half (104; 49.5%) of the respondents had chronic diseases other than diabetes and were taking medications other than for diabetes (**Table 2**).

**Table 2:** Medical conditions of adult male patients with diabetes attending follow-up at Hiwot Fana Comprehensive Specialized University Hospital, Harar, Eastern Ethiopia, 2020 (n = 210).

Variables		Mean(SD)	
Mean SBP			128.8 (**±** 15.8 SD)
Mean DPB			78.6 (**±** 10.3 SD)
Mean weight in kilograms			72 (**±** 9.20 SD)
Mean height in meters			1.72 (**± 0**.042 SD)
**Variable**		**Frequency**	**Percentage (%)**
History of chronic diseases			
Yes		104	49.5
No		106	50.5
BMI			
<18.5		6	2.9
18.5–24.9		103	49.0
25–29.9		93	44.3
>30		8	3.8
Blood pressure			
Normal		117	55.7
Abnormal		93	44.3
Glycemic control			
Good		142	67.6
Poor		68	32.4
Duration of DM since diagnosed			
<5		114	54.3
5–10		61	29.0
>10		35	16.7
Takes medication other than DM			
Yes		104	49.5
No		106	50.5

### Erectile dysfunction among study participants

The magnitude of ED in this study was found to be 83.8% (95% CI: 78.1, 88) of which 37.5% had mild to moderate ED and only 14 (7.95%) had ever sought treatment for the problem (**Table 3**).

**Table 3:** Magnitude of erectile dysfunction among adult male patients with diabetes attending follow-up at Hiwot Fana Comprehensive Specialized University Hospital, Harar, Eastern Ethiopia, 2020 (n = 210).

Variable		Frequency	Percentage (100%)
Erectile dysfunction			
Yes		176	83.8
No		34	16.2
Category of erectile dysfunction (N = 176)			
Severe		12	6.8
Moderate		51	29.0
Mild to moderate		66	37.5
Mild		47	26.7
Ever seek medical care for ED (N = 176)			
Yes		14	7.95
No		162	92.05

### Factors associated with erectile dysfunction among patients with diabetes

Factors associated with ED among patients with diabetes attending follow-up were assessed in the current study. Independent variables in the bivariable logistic regression analysis that had a p value of less than 0.2 were passed for inclusion in the multivariable logistic regression analysis. Age and glycemic control were identified as the independent predictors significantly associated with ED. Adult male patients with diabetes who were more than 60 years old were 2.9 times more likely to experience ED compared with those in the age category of 18–30 years [AOR: 2.9; 95% CI (1.48, 5.67)]. Participants who were in the age category of 46–59 years were 2.56 times more likely to have ED than those who were in the age category of 18–30 years [AOR: 2.56; 95%CI: (1.73, 6.53)]. Likewise, adult male patients with diabetes who had poor glycemic control were 2.14 times more likely to have ED than those who had good glycemic control [AOR: 2.140; 95% CI (1.9, 7.44)] (**Table 4**).

**Table 4:** Multivariable logistic regression analysis for factors associated with erectile dysfunction among adult male patients with diabetes attending follow-up at Hiwot Fana Comprehensive Specialized University Hospital, Harar, Eastern Ethiopia, 2020 (n = 210).

Explanatory variables	Erectile dysfunction		COR (95% CI)	AOR (95% CI)	P-value
	Yes	No			
Educational status					
Illiterate	29	4	4.09 (1.177–14.262)	2.58 (0.501–10.97)	0.089
Elementary (1–8)	77	9	4.835 (1.83–12.74)	6.061 (0.28–12.846)	0.247
High school (9–12)	47	8	3.32 (1.20–9.13)	3.539 (0.521–8.2)	0.146
College and above	23	13	1	1	
Age (years)					
18–30	6	2	1	1	
31–45	21	29	0.24 (0.005–0.087)	0.32 (0.4–2.3)	0.06
46–59	71	8	2.953 (0.125–7.56)	2.560 (1.73–6.53)*	0.004
≥60	65	8	2.7 (0.125–4.756)	2.9 (1.48–4.67)*	0.001
Income					
Less than 1,500 EBR	12	9	0.316 (0.102–0.977)	0.072 (0.002–2.960)	0.166
1,501–3,000 EBR	126	16	1.865 (0.763–4.558)	1.651 (0.108–3.928)	0.640
More than 3,000 EBR	38	9	1	1	
Glycemic control					
Good	112	30	1		
Poor	60	8	2.008 (0.079–6.92)	2.140 (1.9–7.44)*	0.023
Regular physical exercise					
Yes	30	14	0.294 (0.133–0.645)	0.491 (0.513–0.932)	0.175
No	146	20	1		
Chewing khat					
Yes	107	14	2.215 (1.050–4.676)	2.368 (0.056–2.426)	0.299
No	69	20	1		
Duration of DM since diagnosis					
<5	86	28	0.396 (0.129–1.221)	0.159 (0.2–2.438)	0.267
5–10	59	2	3.806 (0.660–21.952)	3.581 (0.03–11.295)	0.720
>10	31	4	1	1	

*statistically significant at p < 0.05; COR, crude odds ratio; CI, confidence interval: AOR, adjusted odds ratio.

## Discussion

The current study assessed the magnitude and factors associated with ED among male patients with diabetes attending follow-up at HFCSH. The study found that an overall magnitude of ED was 83.8%. The finding is comparable with studies conducted in China (14), King Saudi University-Medical City in Saudi Arabia (14), and Bahirdar in Ethiopia (24), which showed ED prevalence of 79.1%, 80.5%, and 85.5%, respectively. However, The finding is comparable with studies conducted in Iran, 59.5% (20); Srilanka, 68% (16); Jamaica, 64% (17); Tanzania, 55.1% (15); Jimma, Ethiopia, 6.8% (21); and Central and Northwestern of Tigray, Ethiopia, 69.9% (19). This inconsistency might be due to sociocultural difference among the study population that talking about sexual issue is not such embarrassing in Iran, Srilanka, and Tanzania as it is in our country, so the population in those studies might get diagnosed and treated accordingly before the study was conducted; different data collection methods and tools to determine the magnitude of ED might be the other reason for the discrepancy that the study that was conducted in the Jimma, Ethiopia, used card review, but this study used interview.

The result of the present study presented that age was significantly associated with ED. Adult male patients with diabetes who were more than 60 years old were 2.9 times more likely to experience ED than those in the age category of 18–30 years, and those participants who were in the age of 46–59 years were 2.56 times more likely to have ED that those who those in the age category of 18–30 years. This current finding is consistent with studies conducted in Turkey (9) and Dessie in Ethiopia (8), where male patients with diabetes whose age were greater than 60 years were more likely to experience ED than those who were less than 60 years old. Old age was also associated with ED among male patients with diabetes in Mizan-Tepi University Teaching Hospital and Tepi General Hospital, Ethiopia (23), and Jimma Medical Center, Southwest Ethiopia (11), where the prevalence was higher among patients greater than 40 years of age than those who were less than 40 years old. Older age was also associated with ED in a study conducted in Bahir-dar Ethiopia where both age group 45–59 and >60 years were associated with ED (24).

Glycemic control is another independent predictor in the present study that exhibited significant association with ED. Adult male patients with diabetes who had poor glycemic control were 2.14 times more likely to experience ED compared to those who had good glycemic control. This is consistent with studies carried out in Italy (10) and Saudi Arabia (9), which revealed a significant association of ED with poor glycemic control. However, in the studies conducted in Turkey (13), Northern Srilanka (16), southwest Ethiopia (23), and Jimma Medical Center Ethiopia (11), there was no association found between ED and glycemic control among adult male patients with diabetes. This variation might be due to differences among the study population, the methodology used, time of study, and diverse population culture.

### Strength and limitation

This study has established some important points that will help us generate a hypothesis. It showed the magnitude of ED among patients with diabetes, which increases from time to time and needs attention. It was also used to see the relationship between the factors and ED among patients with diabetes. Because this study used a cross-sectional study design, cause-and-effect relationship cannot be reported.

## Conclusion

The overall finding of the current study revealed a high magnitude of ED among male patients with diabetes. Majority of the participants experienced mild to moderate ED. Health institutions and healthcare providers should include assessment and management of ED as part of routine medical care in diabetic follow-up clinics. Patients who are of old age and who had poor glycemic control require special attention in screening for ED.

## Data availability statement

The original contributions presented in the study are included in the article/supplementary material. Further inquiries can be directed to the corresponding author.

## Ethics statement

Ethical clearance was obtained from Institutional Health Research Ethical Review Committee (IHRERC) of the College of Health and Medical Science of Haramaya University. The ethical and supportive letters were submitted to Hiwot Fana Comprehensive Specialized Hospital, and consent was obtained from hospital administrator before data collection. A brief introductory orientation was given to the study participants prior to data collection, and written informed voluntary consent was obtained. Moreover, to maintain privacy, the names of the patients were not written on the questionnaire, and patients were interviewed alone in a separate room.

## Author contributions

All the authors had made significant contribution in idea generation, study design, analysis, and interpretation. They participated in drafting and reviewing the manuscript. All authors contributed to the article and approved the submitted version.
